# Supplementary material for: Content-rich biological network constructed by mining PubMed abstracts
Source: BMC Bioinformatics. 2004 Oct 8;5:147. doi: 10.1186/1471-2105-5-147 (PMC528731; doi:10.1186/1471-2105-5-147)
Supplement: Additional File 5 — The original Chilibot query results of the term "long-term potentiation (LTP)" and 22 other terms, limiting the latest references analyzed to the years 1990, 1995, 2000, and 2004. [file 1471-2105-5-147-S5.bz2 › chilibotAdditionalFile5/ltp2004/html/PLC_PI-3K.html]

 


 **PLC** and **PI-3K** 
  
Found 124 abstracts in PubMed,  **30 abstracts were retrieved and analyzed**.  


---

 Search Google  |
 PDF files only 
|  EDU domain only 

---

**Interactive relationship** (e.g. stimulation, inhibition, etc)

- PI3 kinase  [ **PI-3K** ]  products could induce  **PLC**  gamma activation, whose hydrolytic activity could activate the DAG dependent isoforms PKC alpha, theta, and epsilon.  Ref: 14563700 Endocrinology, 2004
- Further studies, conducted in mast cells derived from the bone marrow of mice deficient in the p85 alpha and p85 beta subunits of PI 3 kinase  [ **PI-3K** ] , also revealed no defects in Fc epsilon RI mediated  **PLC**  gamma 1 activation.  Ref: 13129935 J Biol Chem, 2003
- These results suggest that in addition to the positive effects mediated by PI3 kinase  [ **PI-3K** ]  that are apparent in Gab2 mast cells, Gab2 by interacting with Lyn and  **PLC**  gamma may have negative regulatory effects on Fc epsilon RI induced mast cell signaling and functions.  Ref: 11971018 J Immunol, 2002
- In primary cultures of human mast cells, Fc epsilon RI aggregation induced a rapid translocation and phosphorylation of  **PLC**  gamma 1, and subsequent inositol trisphosphate IP3 production, which preceded PI 3 kinase  [ **PI-3K** ]  related signals.  Ref: 13129935 J Biol Chem, 2003
- Here we report evidence that the  **PLC**  gamma 1 dependent pathway of Fc epsilon RI mediated activation of mast cells is independent of PI 3 kinase  [ **PI-3K** ]  activation.  Ref: 13129935 J Biol Chem, 2003
- These results suggest that both diacylglycerol sensitive PKC, activated by  **PLC**  products, and diacylglycerol insensitive PKC, possibly activated by a G i PI 3 kinase  [ **PI-3K** ]  dependent process, are involved in ET 1 induced ERK activation.  Ref: 12055094 Am J Physiol Cell Physiol, 2002
- In addition, although PI 3 kinase  [ **PI-3K** ]  mediated responses were completely inhibited by wortmannin, even at high concentrations, this PI 3 kinase  [ **PI-3K** ]  inhibitor had no effect on parameters of Fc epsilon RI mediated  **PLC**  gamma activation, and had little effect on the initial increase in intracellular calcium levels that correlated with  **PLC**  gamma activation.  Ref: 13129935 J Biol Chem, 2003
- From receptor ligand interactions, activation of heterotrimeric G proteins, their downstream effectors  **PLC**  and PI 3 kinase  [ **PI-3K** ] , the activation of small GTPases of the Rho family, and their regulation of particular cytoskeletal regulatory proteins, we describe pathways specific to the chemotaxing neutrophil and elements documented to be important for neutrophil function.  Ref: 12090462 Crit Rev Oral Biol Med, 2002
- Moreover,  **PLC**  was found to act upstream of the PI 3 kinase  [ **PI-3K** ]  IA isoform.  Ref: 11688990 Biochem Biophys Res Commun, 2001
- U73122, the  **PLC**  inhibitor, effectively inhibited the AA stimulated respiratory burst and calcium release from the intracellular calcium store but not the activity of PI 3K  [ **PI-3K** ] , indicating the independence of PI 3K  [ **PI-3K** ]  signaling on  **PLC**  activation.  Ref: 12949247 J Leukoc Biol, 2003
- The nuclear  **PLC**  gamma1 PIKE S PI 3 kinase  [ **PI-3K** ]  signaling pathway seems to be an extension of the crosstalk between cytoplasmic  **PLC**  gamma1 and PI 3 kinase  [ **PI-3K** ] .  Ref: 14676271 J Cell Sci, 2004
- Results showed that either U73122 or wortmannin inhibited  **PLC**  gamma1 and  **PLC**  gamma1 cells in terms of EGF induced DNA synthesis, cloning formation and cell viability, but  **PLC**  gamma1 cells were more dependent on PI 3K  [ **PI-3K** ]  and less dependent on  **PLC**  compared with wild types.  Ref: 12035061 Sheng Wu Hua Xue Yu Sheng Wu Wu Li Xue Bao (Shanghai)Sheng Wu Hua Xue Yu Sheng Wu Wu Li Xue Bao (Shanghai), 2004
- We conclude that the synergistic interaction between 5 HT and ionophore may be mediated by activation of  **PLC**  calcium and PI 3 kinase  [ **PI-3K** ]  signalling pathways, but definitive proof will require other enzyme inhibitors with greater specificity.  Ref: 11720080 Life Sci, 2001
- These data are consistent with the conclusion that the  **PLC**  gamma dependent component of Fc epsilon RI mediated calcium flux leading to degranulation of mast cells is independent of PI 3 kinase  [ **PI-3K** ] .  Ref: 13129935 J Biol Chem, 2003
- Pharmacological inhibitors of PI3K and  **PLC**  and stable transfection leading to a dominant negative PI3 kinase  [ **PI-3K** ]  construct in transformed fibroblasts abolish macropinocytosis, demonstrating that both enzyme activities are essential.  Ref: 11890548 Int J Med Microbiol, 2002
- Wortmannin, the PI 3K  [ **PI-3K** ]  inhibitor, at the concentration sufficient to inhibit PI 3K  [ **PI-3K** ]  activity, can only partially inhibit calcium release from the internal store, indicating a partial regulation of  **PLC**  signaling by PI 3K  [ **PI-3K** ]  and the existence of two pathways initiated by different  **PLC**  subfamilies.  Ref: 12949247 J Leukoc Biol, 2003

**Parallel relationship** (e.g. studied together, co-existance, homology, etc.)

- Using immunoprecipitation Western immunoblotting however, we were unable to show any direct association between CD4 and  **PLC**  gamma, PI 3K  [ **PI-3K** ] , or other known signaling proteins.  Ref: 12213222 Exp Cell Res, 2002
- The  **PLC**  gamma1 signaling pathway of  **PLC**  gamma1 cells might be complemented by PI 3K  [ **PI-3K** ]  pathway, because after EGF stimulation, the tyrosine phospholation of p85alpha PI 3K  [ **PI-3K** ]  increased significantly in  **PLC**  gamma1... as Western blotting showed that there was neither complementary  **PLC**  gamma2 expression in  **PLC**  gamma1 cells, nor other  **PLC**  isozymes such as  **PLC**  beta and  **PLC**  delta.  Ref: 12035061 Sheng Wu Hua Xue Yu Sheng Wu Wu Li Xue Bao (Shanghai)Sheng Wu Hua Xue Yu Sheng Wu Wu Li Xue Bao (Shanghai), 2002
- The pathway linking  **PLC**  coupled receptors to PI 3 kinase  [ **PI-3K** ]  was deduced to involve phosphoinositide hydrolysis and calcium dependent ErbB3 transactivation but not protein kinase C on the basis of the following evidence i inhibition of carbachol stimulated  **PLC**  by pretreatment with the phorbol ester phorbol 12 myristate 13 acetate concomitantly reduced PKB activity.  Ref: 11694521 J Biol Chem, 2002
- The present study suggests that the  **PLC**  signaling pathway, which may be activated by  **PLC**  beta and  **PLC**  gamma, respectively, and the PI 3K  [ **PI-3K** ]  signaling pathway are involved in the AA stimulated respiratory burst in human neutrophil.  Ref: 12949247 J Leukoc Biol, 2003
- We showed that two inhibitors of Src kinases, PP1 and PP2, abolish both  **PLC**  gamma 2 tyrosine phosphorylation and PI 3 kinase  [ **PI-3K** ]  activity in WT Epo R FDC P1 cells.  Ref: 12504103 Biochem Biophys Res Commun, 2003
- Our results suggest that the Src kinase Lyn is involved in  **PLC**  gamma 2 phosphorylation and PI 3 kinase  [ **PI-3K** ]  activation induced by Epo.  Ref: 12504103 Biochem Biophys Res Commun, 2003
- Phospholipase C gamma1  **PLC**  gamma1 and phosphatidylinositol 3 kinase PI 3K  [ **PI-3K** ]  play crucial role in growth factor induced cell growth and proliferation.  Ref: 12035061 Sheng Wu Hua Xue Yu Sheng Wu Wu Li Xue Bao (Shanghai)Sheng Wu Hua Xue Yu Sheng Wu Wu Li Xue Bao (Shanghai), 2003
- 2 gliclazide treatment produces a gradual translocation of the diacylglycerol DAG dependent isoforms protein kinase C PKC alpha, theta, and epsilon from cytosolic to membrane fraction that is dependent on PI3 kinase  [ **PI-3K** ]  and phospholipase C  **PLC**  gamma activation.  Ref: 14563700 Endocrinology, 2004
- To define the role of phospholipase C  **PLC**  and phosphatidylinositol 3 kinase PI 3K  [ **PI-3K** ] , signaling pathways in arachidonic acid AA stimulated respiratory burst in human neutrophils, the AA stimulated respiratory burst, Ins, 5 P 3 production, PI 3K  [ **PI-3K** ]  activation, and cytoplasmic calcium mobilization were investigated.  Ref: 12949247 J Leukoc Biol, 2003
- Mast cell degranulation following Fc epsilon RI aggregation is generally believed to be dependent on phosphatidylinositide 3 kinase PI 3 kinase  [ **PI-3K** ]  mediated phospholipase C  **PLC**  gamma activation.  Ref: 13129935 J Biol Chem, 2003
- We also demonstrate that the signaling cascade triggered by TNF alpha is governed by the PI 3 kinase  [ **PI-3K** ] , Cdc42 Rac1 and  **PLC**  gamma1.  Ref: 14699068 Mol Biol CellMol Biol Cell, 2003
- Inhibitors to the epidermal growth factor receptor EGFR PD153035, phospholipase C  **PLC**  U73122, extracellular signal regulated kinase ERK mitogen activated protein kinase MAPK PD089035 and PI 3 kinase  [ **PI-3K** ]  Wortmannin were not informative.  Ref: 12115496 Int J Cancer, 2002
- To investigate the complementary mechanism of  **PLC**  gamma1 in cell growth and epidermal growth factor EGF induced mitogenic signaling,  **PLC**  gamma1 deficient mouse embryonic fibroblasts  **PLC**  gamma1 and its wild type  **PLC**  gamma1 were exposed to U73122, a phospholipase C specific inhibitor, or wortmannin, a PI 3K  [ **PI-3K** ]  inhibitor then the clonogenicity, viability, EGF induced DNA synthesis of the two cell lines were determined by cloning formation, MTT method and 3 H thymidine incorporation assay.  Ref: 12035061 Sheng Wu Hua Xue Yu Sheng Wu Wu Li Xue Bao (Shanghai)Sheng Wu Hua Xue Yu Sheng Wu Wu Li Xue Bao (Shanghai), 2002
- We also demonstrated that Epo phosphorylated Lyn is associated with tyrosine phosphorylated  **PLC**  gamma 2 and PI 3 kinase  [ **PI-3K** ]  in WT Epo R FDC P1 stimulated cells.  Ref: 12504103 Biochem Biophys Res Commun, 2003
- Inhibition of phospholipase C  **PLC**  by U 73122 or of phosphoinositide PI 3 kinase  [ **PI-3K** ]  by wortmannin partially reduced ERK activation.  Ref: 12055094 Am J Physiol Cell Physiol, 2002
- The autonomous intranuclear inositide cycle is endowed, besides the  **PLC** , with conventional lipid kinases and phosphatidylinositol 3 kinase PI 3 kinase  [ **PI-3K** ]  which plays an important role in granulocytic differentiation.  Ref: 11729971 Ital J Anat Embryol, 2001
- We identified the 140 and 85 kDa proteins as phospholipase C gamma  **PLC**  gamma and the regulatory subunit of phosphatidylinositol 3 kinase PI 3K  [ **PI-3K** ] , respectively.  Ref: 12213222 Exp Cell Res, 2002
- We examined the role of the Src kinase Lyn in phospholipase C gamma 2  **PLC**  gamma 2 and phosphatidylinositol PI 3 kinase  [ **PI-3K** ]  activation in erythropoietin Epo stimulated FDC P1 cells transfected with a wild type WT Epo receptor Epo R.  Ref: 12504103 Biochem Biophys Res Commun, 2003
- Additional experiments examined the effect of the PKC inhibitor chelerythrine, the  **PLC**  inhibitor U73122, the MAPK inhibitor PD 98059, the G protein inhibitor GDP betaS, the PI 3 kinase  [ **PI-3K** ]  inhibitor LY294002, or the microtubule inhibitor colchicine on EGF induced jejunal glucose transport.  Ref: 11911228 Can J Physiol Pharmacol, 2002
- Thrombin mediated MAPK activation was modulated by PI  **PLC** , calcium, PKC, tyrosine kinase, and PI 3 kinase  [ **PI-3K** ]  associated with cell proliferation in canine cultured TSMCs.  Ref: 11812655 Cell Signal, 2002
- These responses were attenuated by tyrosine kinase inhibitors genistein and herbimycin A, phosphatidyl inositide PI phospholipase C  **PLC**  inhibitor U73122, protein kinase C inhibitor GF109203X, removal of calcium by addition of BAPTA AM plus EGTA, PI 3 kinase  [ **PI-3K** ]  inhibitors wortmannin and LY294002, and inhibitor of MEK1 2 PD98059.  Ref: 11812655 Cell Signal, 2002
- NECA mediated IL 6 release was inhibited by the  **PLC**  inhibitor 1 6 17beta 3 methoxyestra, 5 10 tiene 17 yl amino hexyl 1H pyrrole dione, the PI3 kinase  [ **PI-3K** ]  inhibitor wortmannin and the PKC inhibitors bisindolylmaleimide 1 and bisindolymaleimide X1 HCl Ro 32 0432.  Ref: 14504137 Br J Pharmacol, 2003
